# RNA Splicing Dysregulation in Parkinson’s Disease

**DOI:** 10.3390/genes17080911

**Published:** 2026-07-31

**Authors:** Maria Giusy Bruno, Giacomo Menichetti, Angela Valentino, Aqsa Javaid, Francesca Belpinati, Alessandra Ruggiero, Maria Teresa Valenti, Giovanna Paolone, Fabio Cavaliere, Elisabetta Trabetti, Maria Grazia Romanelli, Cristina Bombieri

**Affiliations:** 1 Department of Neuroscience, Biomedicine and Movement Sciences, University of Verona, 37134 Verona, Italy; mariagiusy.bruno@univr.it (M.G.B.); giacomo.menichetti@univr.it (G.M.); angela.valentino@univr.it (A.V.); aqsa.javaid@univr.it (A.J.); francesca.belpinati@univr.it (F.B.); alessandra.ruggiero@univr.it (A.R.); mariateresa.valenti@univr.it (M.T.V.); elisabetta.trabetti@univr.it (E.T.); 2 Department of Diagnostics and Public Health, Section of Pharmacology, University of Verona, 37134 Verona, Italy; giovanna.paolone@univr.it; 3 Achucarro Basque Center for Neuroscience, The Basque Biomodels Platform for Human Research (BBioH), 48940 Leioa, Spain; fabio.cavaliere@achucarro.org

**Keywords:** splicing, RBPs, RNA processing, Parkinson’s disease, brain organoids

## Abstract

Genetic mutations, altered RNA regulation, and protein aggregation are the main hallmarks of Parkinson’s disease (PD), a neurodegenerative disorder. Investigation into the molecular basis of the disease revealed that post-transcriptional regulation, specifically RNA processing, contributes to neuronal vulnerability in PD. Alterations in alternative splicing affecting genes involved in neuronal function and cellular homeostasis have been reported in PD, including *SNCA*, *LRRK2*, *MAPT*, *PRKN*, and *BIN1*. These alterations impact central neuronal pathways, including cytoskeletal maintenance, mitochondrial function, synaptic activity, oxidative stress, and intracellular trafficking. This review aims to provide an overview of alternative splicing in key PD gene transcripts, with a focus on their roles in pathogenesis and disease progression. Emerging data suggest that dysregulation of RNA binding proteins (RBPs) may influence RNA processing in PD. We will examine current evidence on the RNA regulatory networks in PD, highlighting the role of transcript isoforms and RBPs in neuronal dysfunction. Finally, we will discuss emerging experimental models such as 3D-brain organoids that offer new opportunities to investigate splicing regulation.

## 1. Introduction

Parkinson’s disease (PD) is a neurodegenerative disorder characterized by the gradual degeneration of dopaminergic neurons of the substantia nigra pars compacta (SNPc) [1]. Progressive neuronal loss in the midbrain leads to classic motor symptoms, including bradykinesia, hypokinesia, resting tremor and rigidity, and to non-motor symptoms, including olfactory dysfunction and cognitive impairment, severely impairing the patient’s quality of life [2,3,4]. Over 10 million people worldwide are estimated to be affected by PD, highlighting the need to investigate the molecular mechanisms driving disease onset deeper to find novel therapeutic targets [5]. Mutations in more than 20 genes have been identified as causative of PD, although other factors, such as aging or environmental agents such as pesticides, are strongly correlated with the pathogenesis [6,7]. Several biological pathways contribute to PD pathophysiology, such as cytoskeletal alterations, mitochondrial dysfunction, oxidative stress, and altered vesicle transport [8]. Aberrant protein aggregation and genetic mutations are among the most studied molecular mechanisms, but increasing evidence suggests that RNA post-transcriptional regulation is crucial for PD onset [8,9]. In particular, neurons exhibit more intense RNA processing mechanisms than other cell types, making them more susceptible to deficits in these pathways. RNA metabolism involves transcription, splicing, transport, stability, and translation, thereby making it a highly dynamic and tightly regulated process [10]. Alternative splicing is a key mechanism that modulates the expression of ~95% of human genes, contributing to genomic complexity and proteomic diversity. It generates various mRNA isoforms from a single gene, producing several protein variants with different functions. This process is regulated by a complicated network of RNA-binding proteins (RBPs) called *trans*-acting factors, which bind to specific consensus sequences within pre-mRNAs, called *cis*-acting elements [11,12]. Alternative splicing is a fundamental mechanism that guides neuronal development, and the sequential expression of specific RBPs determines the direction of the cell differentiation. Neuron-specific gene expression guarantees synaptic plasticity, assembly of neural circuits, and cytoskeletal organization [13]. Numerous neurodegenerative diseases, such as PD and Alzheimer’s disease (AD), have been associated with altered RNA splicing, emphasizing the importance of splicing in maintaining neuronal homeostasis [14]. Distinct disease-associated genes are known to undergo the alternative splicing process in the context of PD or neurodegeneration, including α-synuclein (*SNCA*), Leucine-Rich Repeat Kinase 2 (*LRRK2*), Microtubule-Associated Protein Tau (*MAPT*), Bridging Integrator 1 (*BIN1*), and Parkin (*PRKN*), suggesting that splicing dysregulation may affect key biological pathways involved in neuronal survival [15,16,17]. Furthermore, recent studies have shown that dysregulation of RBPs may alter RNA regulatory networks, thus contributing to disease progression [18,19].

**Figure 1:**
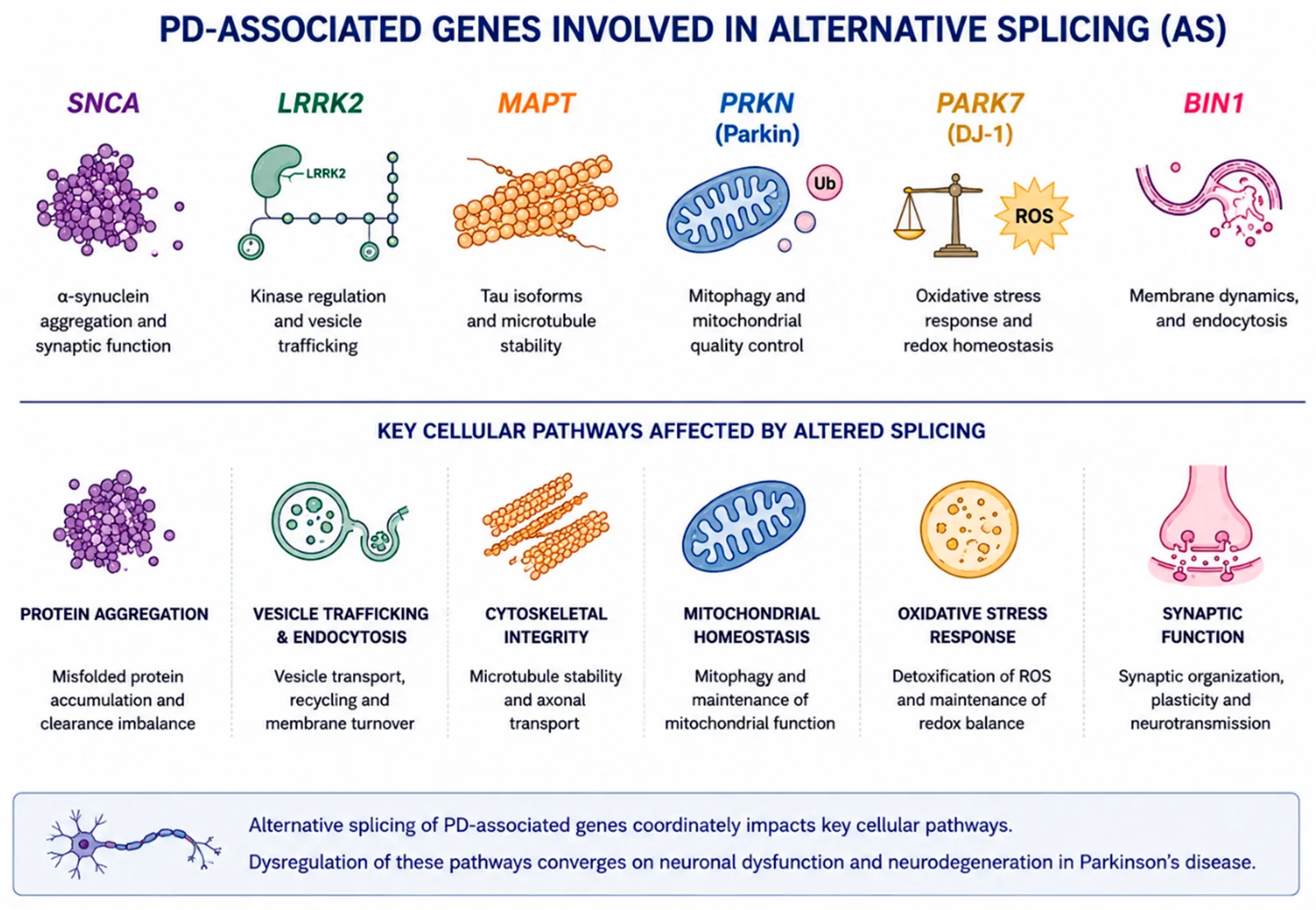
**Overview of PD-associated genes regulated by alternative splicing and the major cellular pathways affected by their dysregulation**. The functional associations shown are supported by the studies discussed throughout this review, including *SNCA* [20,21,22,23], *LRRK2* [24,25], *MAPT* [26,27], *PRKN* [28], *PARK7*/DJ-1 [29,30], and *BIN1* [31,32].

In this review, we provide an updated and critical overview of alternative splicing in Parkinson’s disease, with particular emphasis on PD-related genes whose transcript isoforms have been functionally characterized or are emerging as potential contributors to disease pathogenesis. In addition to established PD-related genes, such as *SNCA*, *LRRK2*, and *PRKN*, we discuss alternative spliced pre-mRNA genes such as *MAPT* and *BIN1*, whose splicing events are correlated to neurodegenerative mechanisms and less investigated in PD. We also examine the contribution of selected RNA-binding proteins, including DJ-1 (gene named *PARK7*), TDP-43, PTBP1 and PTBP2, focusing on the available evidence for their involvement in PD-associated splicing dysregulation. Finally, we discuss emerging human-based experimental models, particularly brain organoids, as promising platforms to investigate disease-relevant splicing alterations in a physiologically relevant context. The main PD-associated genes, RNA-binding proteins, and disease-related cellular pathways discussed throughout this review are summarized in Figure 1.

### Literature Search Strategy

This narrative review was developed based on a comprehensive literature search on alternative splicing and RNA-binding proteins in Parkinson’s disease. The literature search was primarily conducted through PubMed/MEDLINE and complemented, where appropriate, by searches in Scopus and Web of Science, together with manual screening of the reference lists of relevant articles. Search words were “Parkinson’s disease”, “alternative splicing”, “RNA splicing”, “splice variants”, “transcript isoforms”, “RNA-binding proteins”, “PTBP1”, “PTBP2”, “TDP-43”, “DJ-1”, “SNCA”, “LRRK2”, “MAPT”, “BIN1”, “PRKN”, and “brain organoids”. Particular emphasis was placed on recent publications to provide an updated overview of the field; however, seminal studies describing the first identification and functional characterization of specific splice variants were also included whenever necessary to provide historical context and support the interpretation of more recent findings. Peer-reviewed articles and relevant pre-prints were considered when they provided novel evidence that had not yet been incorporated into the published literature. The retrieved studies were critically evaluated and organized into thematic sections covering PD-associated genes, RNA-binding proteins involved in splicing regulation, and experimental models used to investigate splicing dysregulation in Parkinson’s disease.

## 2. Alternatively Spliced Genes Involved in PD

The following sections describe the main genes implicated in PD (Table 1). In particular, we focus on the functions of transcript isoforms generated by alternative splicing, their possible implication in disease-related molecular pathways and the current level of evidence in PD.

### 2.1. SNCA

**Figure 2:**
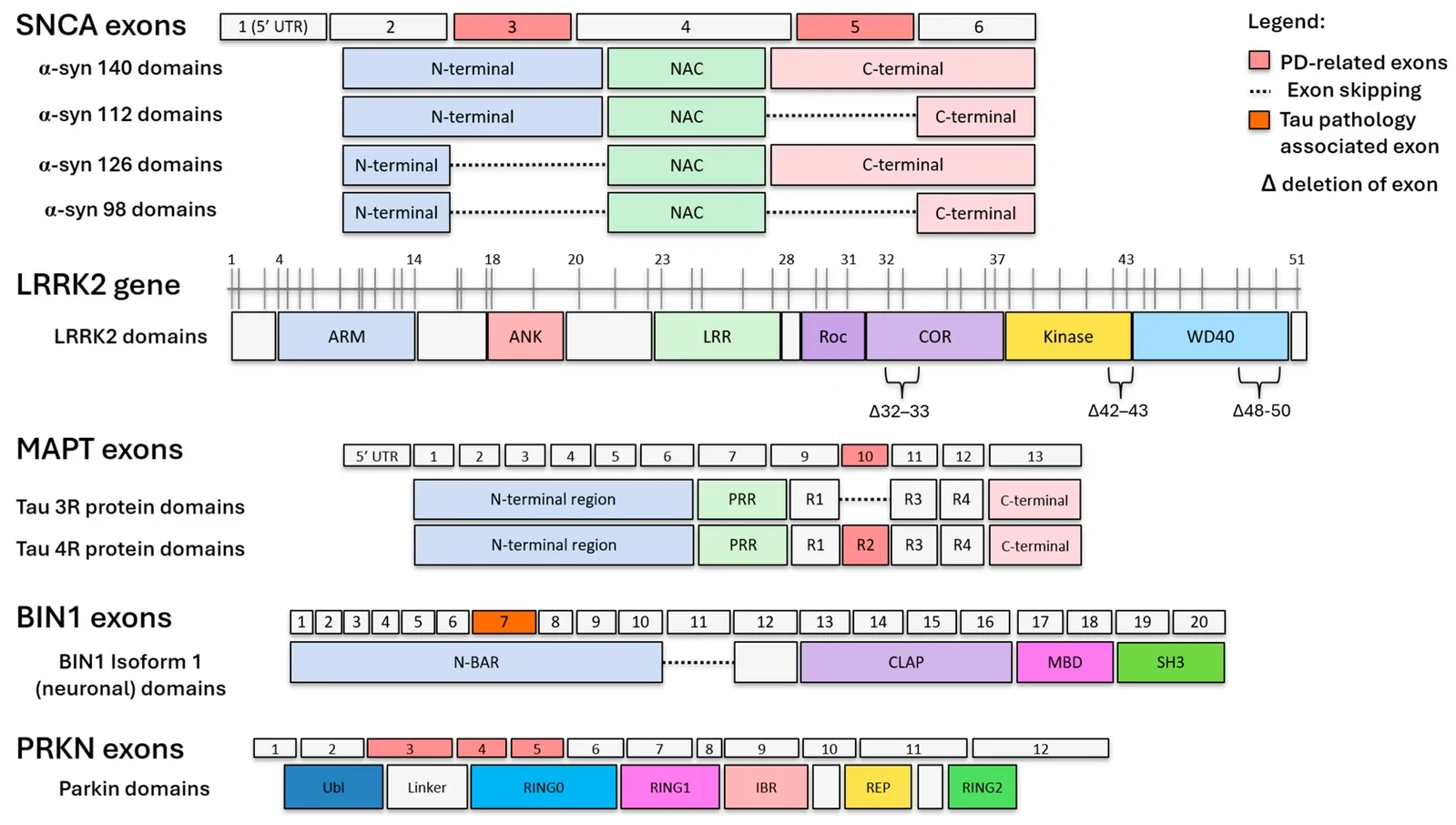
**Schematic representation of the exon organization and protein domain architecture of the major PD-associated genes undergoing alternative splicing**. PD-associated exons are highlighted in red. Protein domains are abbreviated as follows: NAC, non-amyloid-β component; ARM, armadillo repeats; ANK, ankyrin repeats; LRR, leucine-rich repeats; Roc, Ras of complex proteins; COR, C-terminal of Roc; WD40, tryptophan–aspartic acid repeat domain; PRR, proline-rich region; R1–R4, microtubule-binding repeats 1–4; N-BAR, N-terminal Bin/Amphiphysin/Rvs domain; CLAP, clathrin and AP2-binding domain; MBD, Myc-binding domain; SH3, Src homology 3 domain; Ubl, ubiquitin-like domain; RING0/1/2, really interesting new gene 0/1/2 domains; IBR, in-between RING domain; REP, repressor element of Parkin.

α-synuclein is a neuronal presynaptic protein belonging to the synuclein family, characterized by an unfolded structure in physiological conditions. It is a small protein of approximately 15 kDa, involved in the pathogenesis of synucleinopathies, including PD, dementia with Lewy bodies (DLB), and multiple system atrophy (MSA). These disorders present cytoplasmic and neuritic protein inclusions, discovered in 1997 and defined as Lewy bodies and Lewy neurites, which are mainly composed of α-syn aggregates [20,21]. α-synuclein is encoded by the *SNCA* gene that maps to chromosome 4q21.3-q22. According to the Ensembl database annotation, the canonical transcript (ENST00000394991.8, *SNCA*-205) is composed of six exons, of which only five are protein-coding, starting from exon 2. The resulting protein is 140 aa long and is defined as a full-length isoform (α-syn-140) [53]. α-syn-140 isoform is considered the major physiological form of α-synuclein and is predominantly localized at presynaptic terminals, where it binds to vesicle membranes and regulates synaptic trafficking and neurotransmitter release. Structurally, α-synuclein comprises an N-terminal membrane-binding domain, a central non-amyloid component (NAC) region, and a C-terminal acidic domain that lacks a defined structure and modulates protein solubility and aggregation (Figure 2) [54]. Notably, this structural organization is directly affected by *SNCA* alternative splicing transcripts. α-synuclein mRNA undergoes complex alternative splicing mainly involving exons 3 and 5. Alternative in-frame splicing of either exon 3, exon 5 or both results in the formation of three additional isoforms, respectively: α-syn-126, α-syn-112, and α-syn-98 [21,53]. Exon 3 skipping removes 14 amino acids from the N-terminal region, reducing the membrane-binding capacity of the resulting isoform. Otherwise, exon 5 skipping truncates the acidic C-terminal domain, decreasing the number of negatively charged residues and thereby increasing aggregation propensity. Simultaneous skipping of exons 3 and 5 combines both structural alterations in the α-syn-98 isoform (Figure 2) [21].

Early studies on SNCA isoforms expression in PD patients demonstrated that these splice variants are differentially expressed across distinct brain regions. In particular, increased levels of α-syn-140 and α-syn-126 transcripts were detected in the SNPc of PD patients compared to controls. In contrast, α-syn-112 and α-syn-98 showed region-specific upregulation within both PD and control groups [33]. Further evidence identified disease-specific SNCA splicing profiles across different synucleinopathies. In PD, all major α-synuclein isoforms were generally upregulated, with particularly high levels of α-syn-126. In contrast, the pure form of DLB was characterized by increased α-syn-112 and α-syn-98, accompanied by reduced α-syn-140 and α-syn-126 expression [35].

However, in 2018, Vinnakota et al. described a novel 41aa truncated isoform, identified as α-syn-41, resulting from skipping exons 3 and 4 [34]. In this work, brain tissues of PD patients were analyzed for α-syn-140, α-syn-112, and α-syn-41 expression. In the SNPc, all three isoforms were detected as showing little difference in expression, whereas in the cerebellum, α-syn-140 was the most prominent transcript. Characterization of α-syn-41 revealed that it has no propensity for self-aggregation, does not enhance the seeding capacity of other α-syn isoforms, and does not affect cell viability. Nevertheless, cells treated with this truncated peptide exhibited a strong increase of nearly 40% in cellular dopamine uptake via the dopamine transporter (DAT) [34]. This work identifies a novel potential player in PD pathology. However, to date, this isoform has been reported by only one research group, highlighting a significant gap in the literature that warrants further investigation.

In contrast, α-syn-112, generated by exon 5 skipping, was first identified in the human fetal brain by Ueda et al. in 1994, and has since been more extensively studied due to its pathological relevance [55]. The biophysical behavior of α-synuclein splice isoforms has been well characterized by Röntgen et al., who observed that α-syn-112 and α-syn-98 show faster aggregation kinetics than the full-length form. Moreover, a low amount of α-syn-112 is sufficient to facilitate the aggregation of α-syn-140, indicating the ability of splice isoforms to actively modulate the assembly and condensation behavior of α-synuclein [22,23]. More recently, the same group demonstrated that amyloid-β (Aβ42), a well-known pathological hallmark of AD, co-aggregates with the three α-synuclein splice isoforms. Aβ42 seeds induce α-syn fibril formation, suggesting a synergistic effect in which amyloid deposition and SNCA alternative splicing cooperatively contribute to disease progression. These findings further support the model in which the dysregulation of α-synuclein splicing represents a key pathogenic event in PD and it is a promising target for future investigation in neurodegenerative disorders [56].

SNCA splice isoforms are relevant not only for their aggregation propensity but also for their immunological effect. Kaul et al. recently showed that α-syn-112 possesses enhanced antigenic potential compared to both the full-length and α-syn-41 isoforms, inducing a stronger T-cell-mediated inflammatory response in vivo. In contrast, α-syn-41 mainly affected motor coordination without significant dopaminergic neuronal loss, which was consistent with previous observations from the same group linking this isoform to altered dopamine uptake through DAT modulation [34,57]. These results further highlight SNCA’s alternative splicing role in contributing to PD pathogenesis through distinct and isoform-specific mechanisms involving aggregation, neurotoxicity, and neuroinflammation.

Despite growing evidence supporting the pathological relevance of α-syn splice isoforms, the trans acting factors regulating SNCA alternative splicing remain poorly understood. In this context, the RNA-binding motif protein X-linked (RBMX) has been identified as a key regulator of exon 5 splicing under Parkinsonian conditions. Using minigene tools of SNCA exon 5 and the Parkinsonian mimetic 1-methyl-4-phenylpyridinium ion (MPP+), the authors demonstrated that oxidative stress promotes exon 5 skipping and, consequently, α-syn-112 aggregation. Interestingly, RBMX overexpression decreased α-syn-112 mRNA levels and reduced aggregate formation, highlighting a potential protective role for this splicing factor in PD-related pathogenic mechanisms [58].

More recently, long-read isoform and single-nucleus RNA sequencing were combined in the analyses of cortical tissues from PD, DLB, and control subjects, revealing an unexpectedly complex SNCA transcript landscape. Hundreds of distinct transcript isoforms were identified, including several novel splicing events and previously uncharacterized protein variants such as α-syn-115. Interestingly, many aberrant transcripts were enriched in distinct neuronal subtypes in PD and DLB samples, supporting the idea that SNCA expression is subjected to extensive cell-specific post-transcriptional regulation. These findings underscore the complexity of SNCA splicing and further support its central role in the onset and progression of synucleinopathies [36,37]. Overall, SNCA alternative splicing has a central role in PD pathogenesis, as multiple studies in patients and functional investigations have demonstrated isoform-specific differences in expression, aggregation, neurotoxicity, and neuroinflammatory responses. However, the regulatory mechanisms controlling SNCA splicing and the precise contributions of individual splice isoforms to the onset and progression of the disease requires further investigation. Identifying the role of trans-acting factors in the molecular mechanisms regulating SNCA alternative splicing will contribute to molecular stratification, biomarker identification and the design of RNA-based therapeutic strategies.

### 2.2. LRRK2

The Leucine-Rich Repeat Kinase 2 gene, located in 12q1.2, consists of 51 exons and spans approximately 144 kb of the genomic region [59]. LRRK2 is a 2527 amino-acid long multidomain protein including N-terminal armadillo (ARM), ankyrin (ANK), and leucine-rich repeat (LRR) domains, as well as a C-terminal WD40 scaffold (Figure 2). These protein domains collectively mediate protein interactions and control the activity of the catalytic core consisting of Roc (Ras of complex proteins), COR (C-terminal of Roc), and kinase domains. The Roc–COR region has GTPase activity and aids in LRRK2 dimerization, while the kinase domain phosphorylates many Rab GTPases involved in intracellular trafficking, vesicle dynamics, and autophagy [25].

LRRK2 regulates multiple cellular pathways, including synaptic vesicle endocytosis, phagocytosis, Golgi network maintenance, protein sorting, autophagy, microglial function, and endolysosomal homeostasis [24]. Its dysregulation contributes to Parkinson’s disease pathogenesis through impaired α-synuclein and Tau regulation, mitochondrial and synaptic dysfunction, and oxidative stress [60].

*LRRK2* pathogenic variants account for 4% to 5% of genetic PD cases and 1% to 2% of sporadic cases [61,62]. The PD-associated mutations occur mostly within exons encoding the Roc (exon 31), COR (exons 34 and 35), kinase (exon 41), and WD40 (exon 48) domains. In particular, PD has been linked to a number of dominantly inherited *LRRK2* mutations, including p.G2019S and p.I2020T in the kinase domain, p.R1441C/G/H and p.N1437H in the Roc domain, and p.Y1699C in the COR region. Together these mutations underscore the critical role of the Roc–COR–kinase catalytic core in PD pathogenesis [63]. p.G2019S, located in exon 41, is the most prevalent pathogenic variant, and acts as a gain-of-function mutation that raises LRRK2 kinase activity. This variant promotes the formation of α-synuclein inclusions in neurons, suggesting a mechanism by which *LRRK2* mutations could accelerate the development of synuclein pathology in Parkinson’s disease [64,65]. In addition, glial-mediated neuroinflammatory responses have been linked to *LRRK2* G2019S missense mutation, suggesting a wider role in PD etiology beyond neuronal dysfunction [66].

Lubben et al. demonstrated that, by selectively inhibiting with the kinase inhibitor MLi-2, the activity of the mutated LRRK2-G2019S, the progression of Tau pathology can be reversed [67]. Another study showed that asymptomatic *LRRK2* mutation carriers exhibit reduced total α-synuclein levels in cerebrospinal fluid (CSF), along with elevated oligomeric α-synuclein and Tumor Necrosis Factor-alpha (TNF-α), highlighting the value of these biomarkers for early PD detection [68].

Twenty-two transcripts of *LRRK2* have been reported on Ensembl, seven of which are protein-coding. Tissue-specific splice isoforms have been reported in healthy human brain regions, whereas alternative splicing of this gene in PD is still poorly understood (Figure 2). Notably, an isoform lacking exons 32–33, which map to the Roc-COR interface, was identified in both SNPc and the occipital cortex, with greater splice diversity in substantia nigra. Another isoform which skipped exon 32 was observed in the occipital cortex, medulla, and cerebellum. Since the Roc–COR tandem is essential for LRRK2 dimerization and catalytic regulation, these splicing events affect Roc–COR coupling and protein function [38,40]. Similarly, skipping of exons 42–43 removes part of the kinase domain and impairs kinase activity. Moreover, deletion of exons 48–50 found in both SNPc and occipital cortex truncates the WD40 domain, which mediates protein–protein interactions, and therefore is predicted to alter interaction surfaces (Figure 2) [38]. Another study by Giesert et al. has shown the presence of two splice variants in adult mouse tissues. One of the variants with exon 5 spliced out was predominantly expressed in astrocytes, whereas another truncated isoform containing an alternative exon 42a was mainly detected in neurons and astrocytes. This study also suggests that LRRK2 isoforms may contribute to differentially regulated cellular processes in different nervous system cell types [39].

LRRK2 overexpression alters the isoform expression and splicing of key neurodegeneration-related genes, including *MAPT*, *SNCA*, and Dystonin (*DST*) [69]. In addition, LRRK2 was shown to interact with ELAVL4/HuD, an RBP with an emerging role in PD. ELAVL4/HuD regulates the stability, translation, and alternative splicing of important neuronal transcripts, including SNCA, LRRK2, Brain-Derived Neurotrophic Factor (BDNF), Myocyte Enhancer Factor 2C (MEF2C), and Homer Scaffold Protein 1 (HOMER1). LRRK2 directly phosphorylates ELAVL4/HuD and other neuronal ELAV proteins, altering their RNA-binding properties and disrupting normal post-transcriptional regulation, especially mRNA abundance and splicing [70]. In both cellular models and mouse models, the pathogenic *LRRK2* G2019S mutation suppresses ELAV-mediated control of mRNA levels while it enhances ELAV-dependent splicing changes. ELAVL4 knockout (ELAVL4-/-) mice carrying the *LRRK2* G2019S showed α-synuclein and LRRK2 abnormal accumulation, dopaminergic neurodegeneration, and motor impairments. This supports a model in which LRRK2-mediated disruption of neuronal ELAV proteins contributes to pathological features of PD [70]. A noncoding genetic variant located upstream of LRRK2 locus (rs76904798) has also been associated with increased PD risk. Previous studies suggest that this variant may influence LRRK2 expression, further supporting a role for RNA regulatory mechanisms in PD pathogenesis [71].

Current evidence indicates that *LRRK2* variants contributes directly and indirectly to alternative splicing processes. However, direct evidence linking specific *LRRK2* splice isoforms to PD pathogenesis remains limited and presents a need for further investigation.

### 2.3. MAPT

The Microtubule-Associated Protein Tau gene, located on chromosome 17q21, encodes Tau protein, a microtubule-associated protein mainly expressed in the Central Nervous System (CNS) and Peripheral Nervous System (PNS), where it is essential for cytoskeletal stabilization and axonal transport regulation [26].

The gene has 16 exons and shows a complex pattern of alternative splicing involving exons 2, 3, 4a, 6, 8, and 10, producing more than a dozen different Tau protein isoforms [27]. Structurally, the protein is composed of three major domains: the N-terminal projection region (NTR), the proline-rich region (PRR), and the C-terminal microtubule-binding domain (MTBD) (Figure 2). The NTR spans the beginning of the Tau protein to the first residue encoded by exon 7 (amino acids 1–150; numbering refers to the canonical 441-aa CNS Tau isoform). The PRR domain is translated by exons 7 through 9, encoding amino acids 151–243. Finally, the MTBD extends from the remaining portion of exon 9 through exon 12 (amino acids 244–368). In particular, this domain comprises four highly conserved repeats named R1, R2, R3, and R4 [27,72]. Such repetitive peptide sequences modulate axonal transport by directly interacting with tubulin, promoting microtubule stabilization and assembly [73]. In the human adult CNS, six major Tau isoforms are expressed through the alternative splicing of exons 2, 3, and 10. In particular, alternative splicing of exons 2 and 3 generates Tau isoforms with different N-terminal compositions. Inclusion of neither exon gives rise to the 0N isoform and inclusion of exon 2 alone generates the 1N isoform, whereas exon 3 is included only when exon 2 is also present, producing the 2N isoform. Moreover, inclusion or skipping of exon 10 determines the generation of the 4R and 3R Tau isoforms, respectively, by controlling the presence of the second microtubule-binding repeat (R2) within the MTBD (Figure 2) [27,72].

The adult human brain contains approximately equal amounts of 4R and 3R Tau isoforms, which is important for the maintenance of neuronal function. Different mutations inside and in the proximity of *MAPT* exon 10 affect RNA splicing regulatory elements, disrupting the balance between 4R and 3R isoforms [74]. These changes are associated with hyperphosphorylation of Tau, resulting in neurofibrillary tangles, identified in the tauopathies, such as frontotemporal dementia with Parkinsonism-chromosome 17 type (FTDP-17) and AD [75]. FTDP-17 mutations that favor inclusion of exon 10 have been demonstrated to cause overproduction of the 4R Tau isoform. This increased inclusion reflects mutations at the 3′ end of exon 10 that destabilize an RNA stem loop structure that normally promotes efficient splicing of exon 10. Furthermore, cis-acting elements within exon 10 itself are involved in the regulation of splice-site selection. *MAPT* gene mutations affect the alternative splicing of exon 10 by altering enhancer or silencer regulatory elements, leading to either increased or decreased inclusion [76].

In addition to its well-established role in primary tauopathies, a growing body of evidence indicates that MAPT dysregulation is also implicated in PD. The *MAPT* locus has been consistently identified as one of the strongest genetic risk factors for sporadic PD in genome-wide association studies (GWAS) [43,77]. Two extended haplotypes, H1 and H2, are generated by a large genomic inversion at the *MAPT* locus. The H1 haplotype has been consistently associated with an increased risk of PD and other neurodegenerative disorders. Interestingly, the H1 haplotype in mature dopaminergic neurons has been demonstrated to cause specific differences in Tau splicing and functional linking the isoform expression to dopaminergic activity [44]. Specific Tau isoforms have been linked to Parkinsonian phenotypes in diverse studies. Decreased expression of 1N/4R transcript has been correlated with increased bradykinesia and global parkinsonism, while post-mortem studies of PD brains revealed increased accumulation of insoluble 1N4R and 0N3R Tau isoforms [41,45]. Moreover, phosphorylated Tau accumulation has been found in the nigrostriatal system of subjects with early motor impairment and dopaminergic degeneration, even in the absence of α-synuclein aggregates [42]. Other experimental studies have shown that Tau directly interacts with α-synuclein, promoting its aggregation, seeding activity, and propagation. Instead, in vivo Tau depletion reduced alpha-synuclein pathology and Parkinsonian phenotypes [78]. Collectively, this evidence further supports the involvement of Tau-related mechanisms in PD pathogenesis. Alterations in *MAPT* splicing could modulate the aggregation propensity of Tau and its pathogenic interaction with α-synuclein, which might contribute to the progression of PD.

Although the splicing of exon 10 has been studied extensively due to its well-established role in neurodegeneration, little is known about the regulation and functional relevance of the alternative splicing of exon 6. However, Tau isoforms that contain exon 6 have been shown to inhibit Tau polymerization and promote neural differentiation in vitro [79,80]. Recently, we investigated the regulation of Tau protein isoform expression by the splicing factors PTBP1 and RNA-binding motif protein 20 (RBM20). We showed that both RBPs enhance the exclusion of exons 6 and 10 from the mature *MAPT* transcript [81]. However, while the contribution of *MAPT* alternative splicing to primary tauopathies is well established, direct evidence that MAPT splicing dysregulation drives PD pathogenesis remains limited. Clarifying how *MAPT* transcripts influence and contribute to α-synuclein pathology and disease progression will be essential to better define the role of Tau in PD.

### 2.4. BIN1

The Bridging integrator 1 protein was originally discovered in 1996 as a novel interactor of the oncogenic transcription factor c-Myc [82]. Shortly after, BIN1 was identified as a neuronal and muscular protein, also known as amphiphysin II, belonging to the BAR (Bin/Amphiphysin/Rvs) domain superfamily, involved in endocytosis and cytoskeletal regulation [83]. The *BIN1* gene is located on chromosome 2q14.3 and comprises 20 exons that undergo complex cell-type-specific alternative splicing [32,84,85]. In particular, exons 7, 11, 13, 14, 15, 16, and 17 can be retained or skipped, generating at least 14 different transcripts [86]. Exons 1 to 10 encode the N-terminal part of the protein, known as N-BAR, that mediates membrane curvature. Exon 11 corresponds to the phosphoinositide (PI) binding motif, which is present in the skeletal muscle isoforms, mediating the localization in T-tubules. Exons 13 to 16 are brain-specific and encode the CLAP (clathrin and AP2) domain, which regulates endocytosis. Finally, exons 17–18 are translated in the Myc-binding domain (MBD), while constitutive exons 19–20 correspond to a Src homology 3 (SH3) region (Figure 2) [32,85,86]. Overall, the *BIN1* alternative splicing mechanism generates 11 isoforms, of which 1 to 7 are expressed only in the brain, 8 and 12 are muscle-specific, and 9 and 10 are ubiquitous [32,86].

BIN1 was first correlated to neurodegeneration when GWAS identified it as one of the major risk factors for AD, second only to apolipoprotein E (APOE) [87,88]. Since then, extensive studies have sought to elucidate the role of BIN1 in the brain, focusing on the regulation of its isoform expression. In particular, BIN1 isoforms are differentially expressed in neurons, astrocytes, and microglia, highlighting a complex cell-type-specific splicing event [86]. Increasing evidence links BIN1 to Tau pathology and Tau-mediated neurodegeneration. Several studies demonstrated that BIN1 expression affects Tau aggregation, propagation, synaptic localization, and Tau-dependent neuronal dysfunction [89]. Interestingly, reduced inclusion of exon 7, which encodes part of the neuronal N-BAR domain, was associated with increased Tau tangles and cognitive decline, suggesting a protective role of exon 7-containing isoforms against Tau pathology (Figure 2) [86]. Moreover, neuronal BIN1 loss was shown to promote Tau spreading by altering endosomal trafficking and clathrin-mediated endocytosis [31]. BIN1 overexpression promoted neuronal hyperexcitability that was attenuated by Tau reduction, suggesting a functional interplay between BIN1 and Tau-mediated neuronal dysfunction [90]. Altogether, these findings support the idea that dysregulation of *BIN1* alternative splicing may contribute to Tau-mediated neurodegeneration. Although BIN1 has been mainly investigated in AD and tauopathies, where its functional interplay with Tau has been extensively characterized, recent studies have begun to explore whether this pathway may also be relevant in PD. This hypothesis can be supported by the increasing recognition that Tau pathology contributes to disease progression in a subset of PD patients [91,92]. Indeed, genetic studies in PD patients demonstrated a significant enrichment of rare *BIN1* coding variants compared to the controls, suggesting that *BIN1* may also act as a genetic modifier in PD. Interestingly, carriers of *BIN1* variants showed a later age at onset, suggesting a possible modulatory and potentially protective role of specific *BIN1* variants in PD susceptibility and progression [46]. Consistently, similar observations were reported in GBA-associated PD, where the *BIN1* rs13403026 variant was significantly associated with delayed disease onset, further supporting the hypothesis that BIN1 is a genetic modifier in synucleinopathies [47]. Supporting a broader role for BIN1 across α-synuclein-related neurodegenerative disorders, a large GWAS on DLB identified *BIN1* as a major genetic risk loci together with *APOE*, *GBA1* (Glucosylceramidase Beta 1) and *SNCA*, further highlighting the molecular overlap among DLB, AD and PD. RNAseq analyses also revealed that *BIN1* was significantly upregulated in excitatory neurons, while drug–gene interaction analyses indicated it as a potentially druggable target, showing interactions with several known compounds [93].

Despite the growing evidence linking BIN1 to PD susceptibility and neurodegeneration, the contribution of *BIN1* alternative splicing and specific BIN1 isoforms in PD remains largely unexplored. Most available studies have focused on genetic associations or total *BIN1* expression, while the possible role of cell-type-specific splicing alterations in PD pathology still represents an unresolved question. Therefore, whether the BIN1–Tau functional interplay established in AD and related tauopathies contributes to PD pathogenesis requires validations in PD models.

### 2.5. PRKN

The Parkin gene (*PRKN*), located in chromosome 6q25.2–q27, is reported to be implicated in autosomal recessive juvenile parkinsonism (AR-JP) [94], sporadic PD cases [94,95], and less frequent late-onset PD forms [96], but also in other pathological conditions of the nervous system and in cancer [97,98].

The gene, spanning more than 500 kb, is the second-largest gene in the human genome after dystrophin. It is composed of 12 exons and encodes the full-length Parkin protein of 465 aa [94]. The full-length Parkin sequence is characterized by several domains, including the ubiquitin-like domain (UBL) located in the N-terminal region, a cysteine-rich RING0 domain and the two RING1 and RING2 domains separated by an IBR (In-Between RING) domain in the C-terminal region. The UBL confers the E3 ubiquitin ligase status that favors the substrate recognition and its proteasome degradation [99,100], while the IBR play a role in fixing the spatial orientation of the RING domains within the protein (Figure 2) [101]. Alternative splicing modifies the Parkin domain organization by generating protein isoforms with distinct structural architectures. Depending on the skipped exon(s), the resulting proteins may lose or retain the UBL, RING and IBR domains. For example, the skipping of exon 4 produces a truncated protein lacking the C-terminal RING domains, owing to a frameshift and premature stop codon. Otherwise in-frame skipping of exon 5 or exons 3–5 generates shorter isoforms that preserve the C-terminal RING domains but alter the overall domain organization of the protein [49,50].

Parkin is expressed in different tissues, including the brain, heart, testis, and skeletal muscle. In the brain, this gene is expressed in various regions, including the CNS, at both presynaptic and postsynaptic terminals of axons. At the presynaptic terminal, the protein is involved in the release of dopamine. Post-synaptically, Parkin regulates the turnover of postsynaptic density (PSD) proteins, modulating their levels in the synapse [102]. Parkin is involved in numerous molecular mechanisms, including proteasome-dependent degradation of proteins, mitochondrial homeostasis, mitophagy, mitochondrial DNA stability, apoptosis and cell cycle regulation [28,103,104].

According to Ensembl database annotation (*PRKN* ENSG00000185345), the gene may express 24 transcripts, 8 of which are protein-coding, 4 undergo nonsense-mediated decay, 3 are retained-intron transcripts, and the remaining transcripts have incomplete or not fully characterized coding sequence annotations. Different isoforms have been described in the brain and in skeletal muscle [94]. In particular, in patients with AR-JP, two different Parkin isoforms have been reported: one lacking exon 4 and the other lacking exons 3–7 [94]. Sunada et al. described a third isoform from peripheral leukocytes of patients with AR-JP, with the deletion of exons 3–5, producing an isoform of 316 aa, and a fourth one with the deletion of exon 5, expressed in the brain [48]. Interestingly, many of these transcripts maintain the reading frame and preserve the C-terminal RING domains, suggesting that they may encode partially functional proteins rather than loss-of-function products. The functional relevance of these in-frame isoforms is further supported by genotype–phenotype studies showing that AR-JP patients carrying genomic deletions of both PRKN exons 3 and 4 generally exhibit a milder clinical phenotype than those with single-exon deletions. These findings suggest that the internally truncated Parkin protein generated by the combined deletion retains partial biological activity. This provides a rationale for exploring exon-skipping strategies aimed at restoring an in-frame PRKN transcript as a potential therapeutic approach for selected AR-JP patients [105]. These observations indicate that alternative splicing substantially contributes to Parkin protein diversity and may modulate its functional activity [51].

Growing evidence suggests that PRKN splicing alterations may be directly involved in PD pathogenesis. Interestingly, undifferentiated and differentiated SH-SY5Y cells treated with different neurotoxins to mimic the PD-phenotype showed a reduction in the canonical transcript (H1, *PRKN*-206) in both groups and an increase in two alternative isoforms (H20 and H3). This suggests that the splicing mechanism is directly modulated by toxin exposure and that specific Parkin isoforms exert distinct biological functions under pathological conditions [52].

More recently, Lee et al. investigated exon gene expression in familial PD mutations using human pluripotent embryonic stem cells (hPSCs) differentiated into midbrain dopaminergic neurons. The *PRKN* exon 3 deletion revealed 718 altered splicing events compared with isogenic controls. In particular, these changes affected cell projection organization, neuronal differentiation, and cytoskeleton [15]. These results were further supported by the RNA-seq results of the postmortem brain tissue of subjects affected by PD, PD with dementia (PDD), and DLB [106]. These strengthen the idea that abnormalities in pre-mRNA splicing play a significant role in PD pathology. Indeed, transcript isoforms may represent valuable biomarkers for disease detection and progression, as well as promising targets for RNA-based therapeutic interventions [15].

Besides its well-established role as an E3 ubiquitin ligase, accumulating evidence indicates that Parkin regulates gene expression as a transcription factor of p53, presenilin-1 and 2 [107,108]. Ramos et al. have observed in human fibroblasts, in mice, and in sporadic PD-affected human brains that Parkin regulates the transcription of PD genes such as *SNCA* and *GBA1* [109]. These findings reveal an additional mechanism by which Parkin dysfunction may contribute to pathological α-synuclein accumulation and suggest that alterations affecting specific *PRKN* transcripts could have important downstream consequences for proteostasis pathways implicated in PD.

Despite increasing evidence supporting the involvement of PRKN alternative splicing in PD, a comprehensive characterization of Parkin splice isoforms is still limited. The precise exon composition, domain organization, and functional properties of all transcript variants remain poorly defined, making it difficult to establish a unified model of PRKN isoform diversity. Resolving these uncertainties will be essential for understanding the biological significance of individual Parkin isoforms and their contribution to PD pathogenesis.

## 3. RNA-Binding Proteins in Neuronal Homeostasis and Parkinson’s Disease

RNA-binding proteins constitute a broad and functionally distinct class of proteins defined by their capacity to directly interact with RNA through specialized RNA-binding domains, including RNA Recognition Motifs (RRMs), K-homology domains (KH), and Zinc Fingers (ZF) domains [110,111]. These proteins, first well described by Dreyfuss, mediate numerous functions, including alternative splicing regulation, RNA metabolism, transport, localization, mRNA decay, and RNA stability [110,112]. Neurons and glial cells are strongly dependent on regulated RNA metabolism, which is required for brain plasticity [10]. Consequently, perturbation of RBP homeostasis can profoundly affect neuronal survival [10,13]. Given the functional heterogeneity of RNA-binding proteins, the RBPs discussed in this section were selected to represent complementary mechanisms through which RNA metabolism may contribute to PD pathogenesis. PTBP1 and PTBP2 are considered canonical regulators of alternative splicing, whereas TDP-43 and DJ-1 are broader regulators of RNA metabolism whose dysfunction may directly or indirectly affect splicing homeostasis and neuronal survival (Table 2). By including both well-established splicing factors and proteins whose contribution to PD-associated RNA dysregulation is still emerging, we aim to provide a broader perspective on the diverse mechanisms through which RNA-binding proteins may influence PD pathogenesis.

### 3.1. PTBP1 and PTBP2

**Figure 3:**
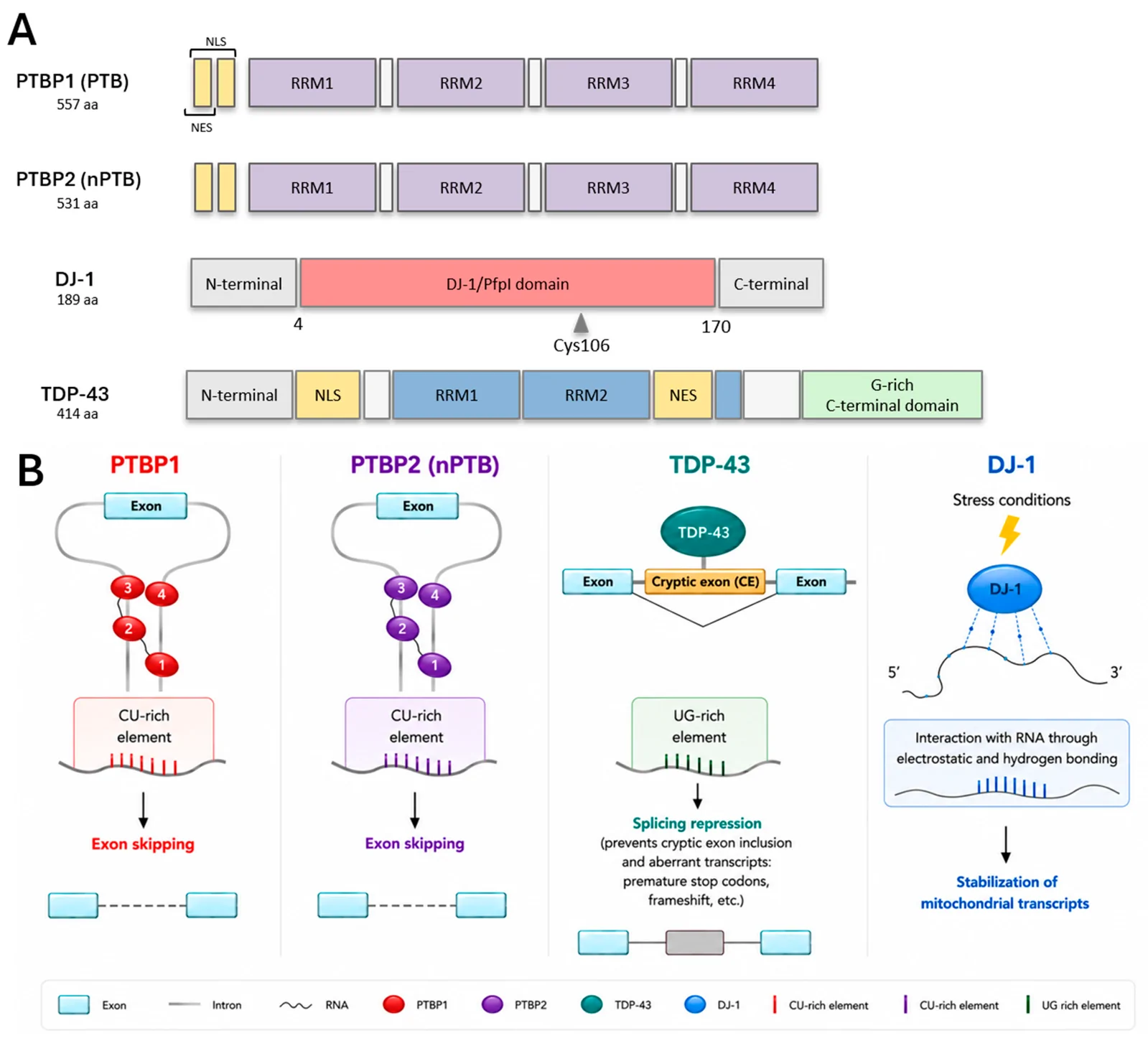
**Structural organization and mechanisms of action of the RNA-binding proteins discussed in this review**. (**A**) Schematic representation of the structural domains of PTBP1, PTBP2, DJ-1 and TDP-43. Protein domains are abbreviated as follows: NLS, nuclear localization signal; NES, nuclear export signal; RRM1–4, RNA-recognition motifs 1–4; PfpI, Pyrococcus furiosus protease I; G-rich, glycine rich domain. (**B**) Overview of the principal mechanisms through which PTBP1 [135], PTBP2 [118], DJ-1 [121,123], and TDP-43 [136] regulate RNA metabolism.

Polypyrimidine tract-binding proteins belong to a highly conserved family of RBPs, primarily known as heterogeneous nuclear ribonucleoproteins (hnRNPs). This family consists of three paralogs named PTBP1, PTBP2 and PTBP3, among which PTBP1 and PTBP2 play particularly important roles in neuronal differentiation [131]. PTBP1 was originally identified through its ability to bind polypyrimidine-rich sequences within precursor mRNAs [132,133]. PTBP1 and its neuronal paralog PTBP2 (nPTB) share a similar structural organization consisting of an N-terminal region containing nuclear localization (NLS) and nuclear export (NES) signals, followed by four RNA-recognition motifs (RRM1–4) responsible for binding pyrimidine-rich sequences such as UCUU and CUCUCU motifs (Figure 3A,B) [134]. Through these interactions, PTBP proteins regulate exon inclusion or exclusion and several post-transcriptional processes, including mRNA stability, polyadenylation, intracellular transport, and cap-independent translation [131].

PTBP1 is ubiquitously expressed across tissues and cell types, whereas PTBP2 is predominantly expressed in the nervous system. High PTBP1 expression characterizes embryonic stem cells and other undifferentiated cellular populations, where it represses neuronal splicing programs to maintain stemness [117,118]. In particular, PTBP1 inhibits PTBP2 expression and prevents the activation of neuronal-specific exons [118,137]. In contrast, during neuronal differentiation, PTBP1 levels decrease progressively while PTBP2 levels increase. This transition is one of the most important molecular events regulating neuronal lineage commitment; therefore, downregulation of PTBP1 allows for the establishment of a neuronal splicing program required for neurite outgrowth, synapse formation, and neuronal maturation [119,137]. Subsequently, PTBP2 regulates a second wave of alternative splicing events necessary for the acquisition of mature neuronal functions. Indeed, even if the two proteins are paralogs and share common targets, they also have distinct non-redundant functions [138].

Given its central role in neuronal identity, PTBP1 has attracted considerable interest as a potential therapeutic target for neurodegenerative diseases. To date, its role in PD is still under investigation. Interestingly, PTBP1 repression was reported to be sufficient to convert astrocytes into dopaminergic neurons capable of restoring the nigrostriatal circuit and improving motor deficits in a mouse model of PD. These findings raised the possibility of exploiting PTBP1 silencing as a regenerative strategy for treating PD [113]. Nevertheless, subsequent studies have strongly contested these results, generating great controversy about the topic. Using astrocyte-specific lineage-tracing approaches, Chen et al. failed to demonstrate conversion of astrocytes into dopaminergic neurons after PTBP1 silencing using adeno-associated virus (AAV) or antisense oligonucleotides (ASO) [114]. However, another group showed that PTBP1 repression in mature neurons still promoted the acquisition of dopaminergic features, increased striatal dopamine levels, and partially rescued motor deficits in PD mice [115]. These discrepancies among studies may arise from differences in astrocyte heterogeneity, disease models, and experimental approaches, indicating that the therapeutic potential of PTBP1-downregulation remains controversial and needs further validation.

Although the involvement of PTBP1 in PD pathogenesis is still poorly understood, its role in the regulation of disease-relevant splicing events has been extensively investigated in Tau-related neurodegenerative disorders like AD. It has been demonstrated that PTBP1 promotes *MAPT* exon 10 skipping, thereby maintaining the balance between 3R and 4R Tau isoforms in the brain [116]. More recently, we have shown that PTBP1 expression correlates with *MAPT* exon 6 alternative splicing, further expanding its role in Tau transcript expression [81]. By contrast, less is known regarding the regulation of PD-associated genes. Transcriptomic analysis reported upregulation of *SNCA* levels upon PTBP1 silencing; however, direct evidence linking PTBP1 to the regulation of specific α-synuclein splice variants is currently lacking [116]. Moreover, indirect evidence suggests a possible role of PTB-family proteins in the regulation of BIN1 transcript expression. In a study investigating the control of *BIN1* exon 7 splicing during cardiac differentiation, Fagg et al. reported enrichment of PTB-family binding motifs and increased PTBP2 expression [139]. Therefore, elucidating the role of PTBP proteins in *SNCA* and *BIN1* splicing may help to clarify the RNA-based mechanisms underlying PD.

### 3.2. DJ-1

DJ-1, encoded by the Parkinsonism-associated deglycase (*PARK7*) gene, is a multifunctional protein highly expressed in dopaminergic neurons present in the substantia nigra. It was first identified as an oncogene involved in the Ras pathway [140] and later as a gene involved in PD [120]. DJ-1 is a 189 amino acid protein that belongs to the DJ-1/ThiJ/Pfpl-like protein family. It is not a classical RNA-binding protein, as it lacks canonical RNA-binding domains such as RRM, KH, or ZF (Figure 3A) [121]. However, it associates with RNA through hydrogen bonding and electrostatic interactions, especially under cellular stress conditions. This interaction occurs between the positive charges on the surface regions of DJ-1 and the negative charges of the RNA backbone (Figure 3B) [121,141].

DJ-1 function impacts several processes, including oxidative stress protection, regulation of mitochondrial function, modulation of metabolism, and regulation of gene expression [19,121,122,142,143]. Indeed, widespread dysregulation of neuronal gene networks involved in synaptic function and stress response has been linked to DJ-1 loss of function [122]. In addition, it has been shown that DJ-1 binds the *FMR1* mRNA, which codify for the Fragile X messenger ribonucleoprotein (FMRP), and upregulates several pathways involved in AD [19]. DJ-1 can also act as a molecular chaperone, preventing protein aggregation and misfolding [144].

It is also reported that DJ-1 prevents misfolding and aberrant aggregation of α-synuclein as DJ-1 knockout facilitates α-synuclein fibril formation [145]. DJ-1 has also been linked to stress-responsive transcriptional programs and RNA-associated regulatory networks involved in antioxidant defense, mitochondrial function, and cell survival [121,122,142]. In particular, DJ-1 exhibits RNA-binding activity under stress conditions and contributes to the stabilization of mitochondrial transcripts. However, it remains unclear whether this activity occurs via direct RNA binding or within RBP complexes [121,123,146].

Loss-of-function mutations in the *PARK7* gene increases the vulnerability of dopaminergic neurons to oxidative stress-induced degeneration, particularly in the substantia nigra. In particular, DJ-1 acts as a redox sensor through the specific residue Cys106, which undergoes oxidation in response to elevated reactive oxygen species (ROS) [29]. This mechanism is particularly relevant in dopaminergic neurons, where the catabolism of dopamine itself is an endogenous source of ROS [147,148]. As a result, in mitochondria, DJ-1 contributes to ROS detoxification; its deficiency enhances mitochondrial fragility and lowers the threshold for stress-induced apoptosis [30].

DJ-1 functionally intersects with the PINK1/Parkin mitophagy axis, and its loss impairs the selective elimination of damaged mitochondria, resulting in their progressive accumulation [30]. Emerging evidence indicates that DJ-1 may also modulate neuroinflammatory responses, including microglial activation and cytokine signaling, suggesting a potential link between oxidative stress regulation and immune pathways in PD [149,150].

In PD, *PARK7* is subject to missense mutations, most of which result in reduced protein stability and impaired antioxidant function. The most notable, L166P mutation, destabilizes DJ-1 homodimer and is associated with early-onset familial PD [151]. However, *PARK7* mutations have been identified in only a limited number of familial early-onset PD cases [152,153], suggesting that DJ-1 may play a broader role in neuronal vulnerability than in monogenic forms of PD. Interestingly, DJ-1 has also been detected within Lewy bodies inclusions in sporadic PD, opening new perspectives for the study of the DJ-1 aggregation mechanism [154]. Oxidative modifications of DJ-1 have been consistently reported in the brains of patients with sporadic PD, indicating that DJ-1 may be functionally affected beyond inherited *PARK7* mutations and may contribute to increased cellular vulnerability in disease-relevant conditions [155,156,157]. Given its central role in oxidative stress responses, mitochondrial homeostasis, and α-synuclein proteostasis, DJ-1 has emerged as a promising therapeutic target in PD. The preclinical approaches focus on small-molecule activators [158], redox-modulating compounds targeting the Cys106-dependent antioxidant activity of DJ-1 [159], and AAV-based genetic strategies targeting DJ-1 in experimental PD models [124].

Although DJ-1 is increasingly recognized as a non-canonical RNA-binding protein, its contribution to RNA metabolism in dopaminergic neurons remains poorly characterized. Further studies are needed to identify its direct RNA targets and determine how DJ-1 RNA-binding functions, together with their impaired oxidative stress defense, contribute to PD pathogenesis.

### 3.3. TDP-43

TAR DNA-binding protein 43, encoded by the *TARDBP* gene, is an RBP involved in splicing regulation and mRNA stability. It is mostly located in the nucleus, but it can also be found in the cytoplasm due to its role in mRNA nuclear import and export [160]. The protein contains functional domains represented by the N-terminal domain (NTD) involved in homodimerization [161], NLS and NES, RRM1 and RRM2 that allow the RNA/DNA binding, and a Glycine-rich low complexity C-terminal domain (LCD) that is involved in protein–protein interaction and in liquid–liquid phase separation (LLPS) (Figure 3A) [162]. TDP-43 dominantly binds RNA in UG-rich sequences and UG-repeats (Figure 3B), which are mostly present in non-coding regions as introns, cryptic exons, 3′UTR, long non-coding RNAs and miRNA precursors, but it could also bind RNA G-quadruplex (rG4) structures, which are mainly distributed in the 3′UTR [163]. The RRM1 domain preferentially binds longer (UG_6_) repeats, and RRM2 binds shorter (UG_3_) repeats [136,162].

TDP-43 is crucial for synaptic plasticity, RNA axonal transport, motoneuron survival, stress response, control of local translation, and mitochondrial metabolism [164,165,166,167]. Because of these roles, TDP-43 strongly impacts cell homeostasis and is closely linked to neuronal degeneration. The role of TDP-43 in splicing is to control exon inclusion/exclusion, pre-mRNA maturation and, mainly, to repress cryptic exon inclusion. Indeed, it has been observed that when TDP-43 is mislocalized to the cytoplasm and depleted from the nucleus, cryptic exons are included in transcripts, leading to widespread disruption of splicing homeostasis [167,168,169,170]. Mutations and loss-of-function of TDP-43 are well-established drivers of Amyotrophic Lateral Sclerosis (ALS) and Fronto-Temporal Dementia (FTD) and have also been implicated in AD and PD [125,171].

Interestingly, TDP-43 pathology appears to be particularly frequent in LRRK2-associated PD. Recent neuropathological studies on brains of both *LRRK2* G2019S and *LRRK2* I2020T mutation carriers reported increased TDP-43 deposition compared with idiopathic PD, partially independent of classical α-synuclein pathology [125,127]. Moreover, brains carrying the *LRRK2* G2019S mutation exhibit significant enrichment of TDP-43-dependent splicing alterations [128]. These findings suggest a tight link between mutant LRRK2, TDP-43 proteinopathy, and RNA splicing dysregulation in PD.

Cytoplasmic mislocalization of TDP-43 in PD brains has also been reported, together with its recruitment into stress granules under oxidative stress conditions [126]. However, TDP-43 pathology is not a defining hallmark of PD, but its presence has been associated with more severe disease progression and increased cognitive impairment [172,173]. TDP-43 inclusions may coexist with classical α-synuclein pathology, suggesting a mixed proteinopathy rather than an exclusively α-synuclein-driven process [174]. Recent evidence suggests that TDP-43 may directly interact with α-synuclein, enhancing its aggregation and toxicity. Consistently, both in vivo and biochemical studies demonstrated that TDP-43 promotes α-synuclein-mediated neurodegeneration and co-aggregates with α-synuclein to form highly toxic hybrid aggregates, supporting a synergistic effect of the two proteins on disease progression [129,130].

In addition, TDP-43 may influence PD pathology by regulating Tau biology. TDP-43 has been shown to regulate MAPT expression by promoting Tau mRNA instability through UG-rich elements located within its 3′ untranslated region (3′UTR). Loss or mislocalization of TDP-43 results in increased Tau expression, suggesting that TDP-43 dysfunction may contribute to Tau accumulation. Although this mechanism has been primarily characterized in tauopathies, it may also be relevant to PD, where Tau pathology is increasingly recognized as a contributor to disease progression [175].

TDP-43 dysfunction has been associated with mitochondrial abnormalities, including increased ROS production and reduced ATP production [176,177]. This aspect is particularly relevant in PD because dopaminergic neurons of the substantia nigra are intrinsically vulnerable to mitochondrial dysfunction. Their high metabolic demand, extensive axonal network, and continuous electrical activity require large amounts of energy, making them particularly sensitive to mitochondrial dysfunction [178]. Furthermore, TDP-43 plays an important role in regulating local protein synthesis at synapses through its association with neuronal messenger ribonucleoprotein granules (mRNPg). TDP-43 proteinopathy impairs granule dynamics, leading to defective mRNA release and reduced local translation, compromising synaptic plasticity and neuronal function [179].

Recent studies report new therapeutic strategies targeting TDP-43 with ASOs. These approaches aim either to modulate *TARDBP* expression directly or to correct downstream splicing defects resulting from TDP-43 dysfunction [180]. Depleting TDP-43 using ASOs or siRNAs to reverse TDP-43-dependent splicing defects in Stathmin 2 (STMN2) and unc-13 homolog A (UNC13A) has emerged as a promising therapeutic strategy for ALS and other related neurodegenerative diseases [181,182]. In parallel, novel therapeutic approaches, such as PROTAC-based methods, are being developed to degrade pathological TDP-43 species selectively [183]. Although these therapeutic strategies are currently being developed primarily for ALS and FTD, they may also represent promising avenues for the treatment of TDP-43-associated pathology in PD. Overall, compelling evidence supports a central role of TDP-43-mediated splicing dysregulation in ALS and FTD, whereas its contribution to PD is emerging, but remains less well defined. Future studies are needed to determine whether TDP-43-dependent splicing alterations actively drive PD pathogenesis or represent secondary events associated with disease progression and proteinopathy.

## 4. 3D Cellular Models to Study PD Pathology and Splicing Events

Organoids are 3D self-organizing structures generated from embryonic stem cells (ESCs) or induced pluripotent stem cells (iPSCs) that mimic the key structural and functional characteristics of the organs [184]. Unlike conventional monolayer cultures, 3D organoids exhibit a greater degree of spatial organization, more realistic cell-to-cell signaling, and an extracellular matrix microenvironment that closely mirrors embryonic development [185]. Despite brain organoid technology being at its beginning and requiring improvement to overcome current structural and biological limitations, such as the lack of microglia [186] and vascularization, brain organoids represent a valuable tool modeling neural development, metabolism, and neurodegenerative diseases [187].

Alternative splicing contributes significantly to the remarkable functional diversity of central nervous system cell types, including neurons and glial cells. Accumulating evidence indicates that dysregulation of this mechanism contributes to the pathogenesis of several neurodegenerative disorders, including PD [14,15]. Consequently, the study of splicing alterations has become an important area of research aimed at understanding the molecular basis of PD and developing novel RNA-based therapeutic approaches. Given the complexity of splicing regulation in the brain, physiologically relevant models are essential. In this regard, organoids have also been used to investigate splicing alterations affecting genes involved in RNA metabolism, cellular stress responses, and protein aggregation pathways [14]. By preserving the patient’s genetic background, the iPSC-derived organoids constitute particularly suitable models for studying alternative splicing and gene expression within a physiologically relevant environment [188].

Among the organoid models, human midbrain organoids (hMOs) derived from iPSCs of PD patients successfully recapitulate the key disease-related pathological features, including impaired neuronal survival, α-synuclein accumulation, reduced dopaminergic function, elevated oxidative stress, mitochondrial impairment, and accelerated dopaminergic cell death [189,190]. Building on these models, patient-derived models carrying mutations in genes such as *SNCA, LRRK2, PINK1* (PTEN induced kinase 1), *PRKN*, and *GBA1* have enabled the identification of molecular pathways involved in neurodegeneration and inflammatory responses [189]. For instance, hMOs with *SNCA* duplications or triplications exhibit a robust, time-dependent accumulation of aggregated α-synuclein, mirroring human autoptic brain pathology more closely than conventional 2D systems [191]. Similarly, organoids generated from patients carrying *GBA1* mutations have revealed defects in neuronal differentiation and alterations in cell proliferation, suggesting a direct role for this mutation in disease pathophysiology [192].

Several studies have demonstrated the utility of organoids for studying alternative splicing across several conditions and tissues. Although PD-specific studies remain limited, organoid models from other neurological disorders have already demonstrated the power of these systems for investigating isoform-specific regulation. For instance, the complex alternative splicing profile of the neurexin 1 (*NRXN1*) gene was studied in hiPSC organoids by an integrative strategy combining single-cell transcriptomics and long-read sequencing, which revealed distinct splicing programs across different neuronal subtypes and glial lineages [193]. Human-stem-cell-derived retinal organoids were studied to identify temporal patterns of isoform usage across developmental stages [194]. In addition, the study of Tau proteoforms, which result from the combination of genetic variations, alternative splicing, and post-translational modifications, across different cellular models, including organoids, revealed a specific pattern of multiple phosphorylation events in advanced AD patients, suggesting a sequential pathway of pathological Tau modification [195]. Similarly, midbrain organoids generated from patients with Progressive Supranuclear Palsy, another tauopathy, recapitulated Tau pathology, including accumulation of the 4R-Tau isoform resulting from exon 10 splicing, supporting the broader relevance of organoid models for studying *MAPT* splicing dysregulation in neurodegenerative disease [196].

As reported above, within the PD framework, genes like *SNCA* and *MAPT* undergo extensive alternative splicing, generating specific protein isoforms with varying propensities for toxic aggregation or enzymatic hyperactivity. In this context, brain organoids provide an ideal model to unravel how the specific splicing events alter cellular homeostasis under chronic conditions [81]. Importantly, Boussaad et al. showed that a *PARK7* mutation impairs U1-dependent splice-site recognition, resulting in abnormal exon 3 skipping, decreased DJ-1 expression, mitochondrial dysfunction, and loss of dopaminergic neurons. Notably, treatment with the kinetin analog RECTAS in combination with phenylbutyric acid rescued the aberrant splicing and reduced neuronal loss in patient-derived brain organoids, providing a proof of concept for precision splicing-correction therapy in PD [197].

Similarly, patient-derived 3D cultures could enable the study of *SNCA* transcript variants, such as the neurotoxic *SNCA-112* isoform, whose altered expression correlates with increased α-synuclein oligomerization and subsequent cell death [21,191]. Additionally, mislocalization or altered activity of specific splicing factors, such as PTBP1 or serine/arginine-rich splicing factors (SRSFs), could also be modeled in hMOs [81]. Patient-derived midbrain organoids carrying the LRRK2 G2019S mutation have been used to evaluate compounds targeting oxidative stress. Homotaurine, a small molecule with reported neuroprotective properties, reduced ROS levels and increased β-catenin expression in these organoids, suggesting a possible strategy for counteracting oxidative stress and impaired signaling associated with G2019S-driven neurodegeneration [198].

The application of bulk RNA sequencing and single-cell RNA sequencing technologies to organoids allows for the identification of differential splicing events associated with specific cell populations and disease stages [199], as well as the investigation of RNA-binding proteins and splicing factors involved in neuronal gene expression regulation and whose dysfunction may contribute to disease progression [112]. An additional advantage of organoid systems is the ability to test therapeutic compounds that correct pathological splicing events directly in patient-derived tissues, thereby facilitating the development of personalized medicine approaches [14]. This strategy has been demonstrated in patient-derived retinal organoids carrying a disease-associated *ABCA4* splice-disrupting variant, where antisense oligonucleotide (ASO) treatment promoted exon inclusion, increased correctly spliced transcripts, and restored protein expression [200]. Notably, the same NRXN1 hiPSC organoid system described above was subsequently used to test splicing correction directly. ASOs targeting mutant splice junctions reduced pathogenic gain-of-function splice isoforms and reshaped splicing patterns in a cell type-specific manner [193]. These findings support the feasibility of testing splice-switching therapeutics for PD genes such as *SNCA* or *PARK7* directly within patient-derived hMOs. Looking ahead, combining brain organoids with single-cell sequencing technologies and genome-editing approaches is expected to accelerate the discovery of novel transcriptomic biomarkers and therapeutic targets for PD treatment [189]. Single-cell resolution together with long-read sequencing in iPSC-derived organoids has already proven powerful for resolving splicing at the level of individual cell types. This combined approach in cerebral organoids identified thousands of previously uncatalogued isoforms and revealed cell-type-specific splicing events, including coordinated splicing and intron-retention patterns that are difficult to detect with short-read sequencing alone [199]. Using similar approaches to hMOs would allow splicing programs in dopaminergic neurons to be distinguished from those in astrocytes, oligodendrocytes, or progenitor populations, refining the cell-type specificity of PD-associated splicing defects and clarifying whether disease-associated changes in genes such as *SNCA*, *MAPT*, and *PARK7* are restricted to cellular populations.

In addition to transcriptomic approaches, CRISPR-generated isogenic organoids offer a powerful strategy to establish genotype–phenotype relationships in PD by minimizing confounding effects from inter-individual genetic heterogeneity. For instance, *SNCA*-triplication midbrain organoids reproduced key synucleinopathy features—α-synuclein aggregation and dopaminergic neuronal loss—that were absent in CRISPR/Cas9-corrected isogenic controls from the same genetic background, confirming these phenotypes as mutation-specific [191]. This strategy could similarly be applied to determine whether specific PD-associated splicing alterations directly drive neuronal dysfunction. In another study, cortical organoids generated from patients carrying the intronic *MAPT* IVS10 + 16 mutation were compared with isogenic control organoids corrected for the mutation. Mutant organoids showed impaired synaptic maturation, reduced neuronal activity, and altered mitochondrial function, phenotypes that were absent in the isogenic controls. Bezafibrate treatment rescued the mutant phenotype, restoring the mitochondrial content and synaptic function [201].

Overall, the use of organoid models to investigate alternative splicing and its deregulation in PD would improve understanding of disease pathogenic mechanisms, supporting the development of new possible patient-specific therapeutic strategies.

## 5. Conclusions

Parkinson’s disease is increasingly recognized as a complex disorder in which genetic mutations, protein aggregation, and changes in RNA metabolism interact to cause neuronal dysfunction and degeneration. Alternative splicing is an important post-transcriptional mechanism that has emerged as a contributor to disease pathogenesis by regulating several PD-associated genes, including *SNCA*, *LRRK2*, *MAPT*, *PRKN*, and *BIN1*. The production of different transcript isoforms can strongly affect protein function, aggregation propensity, mitochondrial homeostasis, synaptic activity, and intracellular trafficking, highlighting the biological relevance of splicing regulation in dopaminergic neurons.

Increasing evidence suggests that RBPs, centrally involved in defining splicing programs such as PTBP1, PTBP2, DJ-1, and TDP-43, may be involved in PD progression. Particularly significant for the future will be to identify the specific RBP-target networks that regulate PD-associated transcripts. The emergence of human iPSC-derived brain organoids and other advanced cellular models now offers unprecedented opportunities to study splicing regulation in a context that faithfully mimics the physiological one. Improved understanding of PD-specific RNA-processing mechanisms could lead new insights into PD pathogenesis and open new avenues for the discovery of novel biomarkers and therapeutic targets for precision medicine approaches.

## Figures and Tables

**Table 1:** Alternative splicing events and major transcript isoforms of PD-associated genes.

Gene	N. of Exons	Major Splicing Events	Main AS Isoforms	Functional Relevance in PD	Type and Current Level of Evidence in PD	Ref.
*SNCA*	6	Skipping of exons 3, 5, 3 + 5, 4	α-syn140, α-syn126, α-syn112, α-syn98, α-syn41, α-syn115	Altered aggregation propensity, Lewy body formation, phase separation and neuronal toxicity	Human PD post-mortem brains, cellular models, biochemical aggregation studies, long-read and single-nucleus RNA sequencing; alternative splicing in PD. Availability of functional isoforms data	[22,23,33,34,35,36,37]
*LRRK2*	51	Splicing involving exons 5, 32–33, 42a, 42–43, 48–50	Full-length kinase-containing isoform; truncated transcripts lacking portions of ROC/COR/kinase domains	Altered splicing may alter kinase activity, GTPase function and intracellular trafficking	Healthy human post-mortem brains (SNPc, occipital cortex, medulla, and cerebellum); adult mouse tissues. Genetic relevance in PD and broader relevance in neurodegeneration	[38,39,40]
*MAPT*	16	Alternative splicing of exons 2, 3, 6 and 10	0N, 1N, 2N isoforms; 3R and 4R Tau isoforms	Tau aggregation, microtubule dynamics, neuronal dysfunction	Human PD post-mortem brains, GWAS, mature dopaminergic neurons. Availability of functional isoforms data. Alternative spliced in PD. Genetic relevance in PD	[41,42,43,44,45]
*BIN1*	20	Inclusion/skipping of exons 7, 11, 13–17	Brain-specific (1–7), muscle-specific (8,12), ubiquitous (9,10) isoforms	Endocytosis, membrane remodeling, Tau pathology and PD susceptibility	Human genetic association studies in PD patients. Broader relevance in neurodegeneration. Genetic relevance in PD	[46,47]
*PRKN*	12	Extensive exon skipping involving exons 3, 4, 5, 7 and combinations thereof	More than 20 transcripts reported; major isoforms include Δex3, Δex3–4, Δex4, Δex5, Δex7 variants	May affect E3 ubiquitin ligase activity, proteostasis and α-synuclein regulation	Human patient samples (AR-JP); cellular models; human pluripotent stem cell-derived dopaminergic neurons; RNA-seq on human post-mortem PD brains. Availability of functional isoforms data	[15,48,49,50,51,52]

**Table 2:** RNA-binding proteins implicated in Parkinson’s disease and their known functions and characteristics.

RBPs	Structural Domains	RNA Motif Recognized	Main Physiological Functions	Evidence in PD	Type and Current level of Evidence in PD	Ref.
PTBP1	4 RRMs, NLS, NES	Pyrimidine-rich motifs (UCUU, CUCUCU)	Alternative splicing, mRNA stability, polyadenylation, RNA transport, IRES-mediated translation, maintenance of stem/progenitor identity, and repression of neuronal splicing programs	Controls the PTBP1/PTBP2 developmental switch during neurogenesis. Proposed target for neuronal reprogramming in PD, although results remain controversial. Regulates MAPT exon 10 and exon 6 splicing, while its role in SNCA and BIN1 splicing remains largely unexplored.	Cellular models; mouse models of PD; transcriptomic studies	[113,114,115,116]
nPTB	4 RRMs, NLS, NES	Pyrimidine-rich motifs	Establishment of neuronal-specific splicing programs, neurite outgrowth, synapse formation, and neuronal maturation	Upregulated during neuronal differentiation and required for neuronal transcriptome remodeling. Potential contributor to PD-associated splicing alterations, although direct evidence is limited.	No direct experimental evidence in PD; proposed role inferred from neuronal splicing programs	[117,118,119]
DJ-1 (*PARK7* gene)	Non-canonical RNA-binding protein (no RRM/KH/ZF domains)	Electrostatic interactions with RNA	Oxidative stress sensing, mitochondrial homeostasis, RNA regulation, transcriptional control, and chaperone activity	Familial early-onset PD gene. Oxidized in sporadic PD and detected in Lewy bodies. May modulate α-synuclein aggregation and stress-responsive RNA networks involved in neuronal survival.	Human genetic and post-mortem studies; cellular and biochemical studies; preclinical experimental models.	[120,121,122,123,124]
TDP-43	2 RRMs, NLS, NES, glycine-rich LCD	UG-repeats and UG-rich motifs ((UG)n, UGUGUG); rG4-structures	Alternative splicing, cryptic exon repression, mRNA transport, local translation, stress granule dynamic, and RNA quality control	Cytoplasmic inclusions reported in a subset of PD cases, particularly those with cognitive impairment. Regulates MAPT mRNA and may interact with α-synuclein, contributing to mixed proteinopathies.	Human post-mortem PD brains; cellular, biochemical and in vivo studies; limited PD-specific preclinical therapeutic evidence	[125,126,127,128,129,130]

## Data Availability

No new data were created or analyzed in this study. Data sharing is not applicable to this article.
